# Use of Heterophilic Blocking Tubes in Suspected Heterophile Antibody Interference Among Pubertal Patients

**DOI:** 10.3390/medicina62010129

**Published:** 2026-01-08

**Authors:** Aysun Ekinci, Revsa Evin Canpolat Erkan, Ismail Yildiz, Naile Fevziye Misirlioglu, Hafize Uzun

**Affiliations:** 1 Department of Biochemistry, Faculty of Medicine, Dicle University, Diyarbakir 21280, Türkiye; drevinerkan@gmail.com; 2 Department of Biostatistics, Faculty of Medicine, Dicle University, Diyarbakir 21280, Türkiye; iyildiz21@yahoo.com; 3 Department of Biochemistry, Faculty of Medicine, Istanbul Atlas University, Istanbul 34403, Türkiye; nailemisirlioglu@gmail.com (N.F.M.); huzun59@hotmail.com (H.U.)

**Keywords:** heterophile antibody, immunoassay, interference, puberty, luteinizing hormone

## Abstract

*Background and Objectives:* Today, immunoassay methods are still widely used in the analysis of hormone tests. Due to the properties of the reagents used in immunoassay analyses and components other than the measured analyte, deviations in clinical results may occur. There are many factors that cause this condition called interference, and one of the most common of these is heterophile antibody (Ab). Puberty is a process that begins between the ages of 8 and 13 in girls and 9 and 14 in boys. Pulsatile luteinizing hormone (LH) release during sleep is the first hormonal change that indicates the approach of puberty. The reliability of the laboratory analysis result is important. In order to determine whether there is a risk of interference in the LH tests we analyzed in our laboratory, 48 serum samples of pediatric patients belonging to the pubertal age group were included in the study. *Materials and Methods:* In order to evaluate the suspicion of heterophile Ab interference, we measured the samples again by binding the antibodies and removing them from the matrix, as recommended in the Clinical and Laboratory Standards Institute (CLSI) I/LA30 guideline. For this, we used a heterophile blocking tube (HBT). We analyzed the samples with Beckman Coulter UniCel DxI 800 and Roche Cobas e601 immunoassay systems. We aliquoted the supernatants of the samples processed according to the HBT application protocol and measured them on both autoanalyzers. *Results:* We found a significant difference between the results of the samples measured before and after HBT pretreatment on the Beckman Coulter UniCel DxI 800 autoanalyzer (*p* = 0.01). LH values after HBT were higher than those before HBT: very high LH values were obtained in 4 patients, while the values showed increases ranging from 2 to 4.64-fold in 5 patients. There was no significant difference between the results evaluated before and after HBT pretreatment on the Roche Cobas e601 autoanalyzer (*p* = 0.27). Although there was a significant difference between the LH results of the HBT-untreated sera obtained in two different autoanalyzers (*p* < 0.001), we found that the LH measurements after HBT pretreatment did not create a statistically significant difference between the two devices (*p* = 0.76). *Conclusions:* We concluded that while HBTs were ineffective in detecting heterophile antibody interference in LH testing, the study underscores the complexity of interference in pediatric hormone assays and highlights the need for further investigation into alternative methods to ensure reliable test results in this age group.

## 1. Introduction

Interference in clinical chemistry is defined as any factor originating from sample components that causes a clinically significant deviation from the true analyte concentration. Such interference may arise from characteristics of the analytical system, suppression of indicator reactions, inhibition of analytes, or the formation of antibodies against assay reagents [1]. Immunoassay methods, which are widely used for the measurement of hormones and other immunological parameters, have evolved with technological advances; however, analytical interferences remain a persistent challenge. Among these, heterophile antibody (Ab) interference is one of the most common causes of erroneous results in immunometric assays [2,3].

Heterophile Abs are polyclonal antibodies capable of binding to antibodies from different animal species used in assay reagents. They typically lead to falsely elevated, and less frequently falsely decreased, test results. The reported prevalence of heterophile antibody interference in immunometric assays ranges from 0.4% to 4%, depending on antibody concentration, binding affinity, assay design, and analytical protocol [1].

Heterophile antibodies may belong to the IgG, IgM, or IgA subclasses. Human anti-animal antibodies (HAAAs), most commonly human anti-mouse antibodies (HAMAs), are monoclonal antibodies that may develop following exposure to animal-derived products or certain infectious agents and can similarly interfere with immunoassays. In addition, rheumatoid factor (RF), an IgM antibody directed against the Fc portion of human IgG, is another well-recognized source of immunoassay interference [2,4].

In pediatric hormone testing, such analytical interferences may result in clinically significant consequences, including misdiagnosis, unnecessary diagnostic procedures, and inappropriate treatment decisions. Puberty represents a complex developmental period characterized by dynamic hormonal, physical, and psychological changes [5,6,7]. Although the precise mechanisms initiating puberty remain unclear, factors such as adiposity, adrenal androgen production, and psychosocial stress have been implicated [8]. Puberty typically begins between 8–13 years in girls and 9–14 years in boys and is marked by increasing gonadotropin secretion following the release of central inhibition of gonadotropin-releasing hormone (GnRH). The earliest hormonal sign of pubertal onset is the emergence of pulsatile luteinizing hormone (LH) secretion during sleep [9].

Adolescents constitute a particularly vulnerable population for analytical interference because physiological hormonal fluctuations are already pronounced during puberty. Even minor assay-related deviations may shift hormone concentrations into pathological ranges, leading to misinterpretation. Therefore, heightened awareness of heterophile antibody interference and appropriate laboratory verification strategies is essential in this age group.

In this study, patients were included after clinicians reported discrepancies between physical examination findings and laboratory results. We focused on LH measurements, which play a critical role in the diagnosis and monitoring of precocious puberty. The aim of this study was to evaluate the potential risk of heterophile antibody interference in LH assays performed during puberty and, when necessary, to inform clinicians prior to result reporting to prevent unnecessary testing, diagnostic delays, and inappropriate clinical management.

## 2. Material and Method

We included 48 serum samples of children aged 8–13 years old in the pubertal age group who applied to the routine outpatient clinic of the University Hospital between 1 December 2024–28 February 2025 in our study. (This study protocol was approved by the Ethics Committee of University, 20 November 2024, decision number 113).

To evaluate the suspicion of heterophilic Ab interference, antibodies were bound and removed from the matrix as recommended by the Clinical and Laboratory Standards Institute (CLSI) I/LA30: Immunoassay Interference by Endogenous Antibodies [10] guideline and measured again in the samples. For this, a heterophilic blocking tube (HBT) (Scantibodies Laboratory, Inc., Sanlee, CA, USA, part number: 3IX762) was used. Serum was obtained from the samples taken into BD Vacutainer SST II Advance gel tube and centrifuged at 4000 rpm for 10 min (Hettich Rotina 380 R, Andreas Hettich GmbH & Co. KG, Tuttlingen, Germany). LH values were analyzed within one hour with Beckman Coulter UniCel DxI 800 (Brea, CA, USA) and Roche Cobas e601 (Roche Diagnostics, Mannheim, Germany) immunoassay systems. After aliquoting the supernatants of the samples treated according to the HBT application protocol, simultaneous LH test measurements were performed on both autoanalyzers. The potential interference of heterophile antibodies in LH assays was statistically evaluated both within individual autoanalyzers and across different autoanalyzer platforms based on the obtained results.

The Beckman Coulter Access human LH (hLH, Lutropin) reagent is a sequential two-step immunoenzymatic (“sandwich”) chemiluminescence assay with paramagnetic particles coated with goat anti-mouse: mouse anti-hLH complexes for the quantitative determination of LH levels. hLH binds to mouse anti-hLH immobilized on solid phase. The material bound to the solid phase is held in a magnetic field while unbound material is washed away. Alkaline phosphatase-conjugated goat anti-hLH is then added and binds to the hLH previously bound to the particles. After incubation, the material bound to the solid phase is held in a magnetic field while unbound material is washed away. The chemiluminescent substrate is then added to the cuvette and the light produced by the reaction is measured with a luminometer. The light production is directly proportional to the analyte concentration in the sample. Measurement range: 0.3–250 mIU/mL [11].

The Roche Elecsys assay kits (Roche Diagnostics GmbH) use two monoclonal antibodies specifically directed against human LH in the LH test. Biotinylated monoclonal anti-LH antibody (mouse) detects the epitope formed by both subunits, while ruthenium complex Tris(2,2′-bipyridyl) ruthenium (II)-complex (Ru(bpy)) labeled monoclonal anti-LH antibody (mouse) detects the epitope formed by the β-subunit. Biotinylated monoclonal LH-specific antibody and ruthenium complex labeled monoclonal LH-specific antibody form a sandwich complex. After addition of streptavidin-coated microparticles, the complex becomes bound to the solid phase through the interaction of biotin and streptavidin. Application of voltage on the electrode induces chemiluminescence emission, which is measured with a photomultiplier. Child reference range: mIU/mL (Girls; 6–10 years: <0.1–3.1; 11–13 years: <0.1–11.9 Boys; 6–10 years: 0.1–1.4; 11–13 years: 0.1–7.8) [12].

The HBT usage procedure was as follows: Before using the tube, it was tapped on a hard surface to ensure that all the reagent inside it reached the bottom. 500 μL of patient sample was placed inside, its cap was closed and turned upside down 5 times. It was incubated for 1 h at 18–28 °C. Then, the same procedure was applied with the other samples, and the LH level was measured in the autoanalyzer.

An HBT contains a single blocking reagent consisting of special connections that inactivate heterophilic antibodies. The reagent in each tube has the ability to inactivate heterophilic antibodies found in 500 μL of sample. The reagent is in the form of lyophilized particles at the bottom of the tube. An HBT replaces a sample pretreatment/secondary test performed to confirm or invalidate the original test result without pretreatment [13].

### Statistical Analysis

The IBM SPSS 21.0 for Windows statistical package program was used in the statistical evaluation of our research data. Measurement variables are presented as mean ± standard deviation (SD), Median, Minimum value, Maximum value, categorical variables are presented as number (n) and percentage (%). Kolmogorov–Smirnov test was used to check whether the data were normally distributed. Wilcoxon Test was used to compare the results of two devices that did not show normal distribution. Hypotheses were taken as two-sided, and *p* ≤ 0.05 was accepted as statistically significant.

## 3. Results

In our study, data of a total of 48 patients, 38 girls and 10 boys in the pubertal age group, were analyzed. Age distribution according to gender is shown in Table 1. Table 2 presents age- and gender-stratified LH concentrations measured using two different immunoassay platforms before and after HBT treatment. We found a significant difference between the sample results measured before and after HBT pretreatment on the Beckman Coulter UniCel DxI 800 autoanalyzer (Brea, CA, USA) (*p* = 0.01) (Table 3). The LH values after HBT pretreatment were higher than the values before HBT: very high LH values were obtained in 4 patients, while the values in 5 patients showed increases ranging from 2–4.64 fold (Table 4). The result of 1 patient was the same, and the results of 5 patients were close to each other (Table 2). There was no significant difference between the results in the evaluation made on the Roche Cobas e601 autoanalyzer before and after HBT pretreatment (*p* = 0.27) (Table 3). Although there was a significant difference between the LH results of the first HBT pretreatment-free sera obtained on two different autoanalyzers (*p* < 0.001), we found that the LH measurements after HBT pretreatment did not create a statistically significant difference between the two devices (*p* = 0.76) (Table 5).

## 4. Discussion

The fast measurement and result delivery time and the analytical performance of autoanalyzers have made immunometric tests indispensable for laboratories. There are two methods frequently used in routine patient analysis today: competitive immunoassays and non-competitive sandwich type/two-site immunoassays [2].

In competitive immunoassay types, since the analyte concentration is lower, heterophile Ab interference is observed more frequently [3]. Both autoanalyzers included in the study employ non-competitive sandwich immunoassay methods.

Clinical laboratories should follow the recommendations of published guidelines to ensure that hormone results obtained using immunoassays are as accurate and precise as possible [2]. The CLSI I/LA30 guideline recommends contacting the manufacturer in case of suspicious patient results and informing them about interference issues [7]. First of all, we suspected our LH results in patients in the pubertal age group after consulting with clinicians and investigating them. The local representative of the Beckman Coulter brand autoanalyzer that we measure in our laboratory was contacted. As stated in the CLSI I/LA30 guideline, it was decided to compare the results by measuring the same samples on a different autoanalyzer (Roche Cobas e601) for method comparison. Heterophilic Ab interference was suspected when there was a significant difference (*p* < 0.01) between the results of the two immunometric devices [1]. To reduce heterophile Ab interference, it is necessary to incubate the reactive antibodies in the patient serum with the same type of serum, so that they will bind and precipitate the heterophile Abs [2]. Nowadays, it is also possible to inactivate them by using commercial heterophile blocking tubes containing specific binders that serve this function [2], and moreover, they provide a more standardized form. Commercial HBT was used in our study for this purpose.

The sample sera were pretreated with HBTs to detect the presence of heterophile Ab. The LH values we measured after the HBT pretreatment in the Beckman Coulter analyzer were higher (*p* = 0.01). We expected that HBT would bind the heterophile antibodies in the matrix and reduce the high LH values. Potential mechanisms for HBT failure in LH tests may include interference from the glycoprotein structure of LH, epitope masking, or reactive components. LH consists of two covalently linked glycoprotein subunits, alpha and beta. LH, with a molecular weight of 28,500 daltons, has two N-linked carbohydrate chains in the alpha subunit and an asparagine-linked oligosaccharide in the beta subunit. The alpha subunit of LH has a similar structure to human chorionic gonadotropin (hCG), FSH, and thyroid-stimulating hormone (TSH) glycoproteins. However, it is the beta subunits that determine immunological and physiological specificity [11,12]. In the manufacturer’s kit limitations section, it is mentioned that the LH test shows <0.1% cross-reactivity with growth hormone (GH) and human placental lactogen (PL) in addition to these hormones [9]. Patients’ LH tests may have cross-reacted with other tests of similar structure.

Takahashi et al. [13] examined the serum sample of a 74-year-old male patient who received an incorrect Graves’ disease diagnosis and treatment (methimazole) due to erroneously high free triiodothyronine (FT4), free thyroxine (FT3) and thyrotropin receptor antibody (TRAb) levels as a result of Elesys assay kits (Roche Diagnostics GmbH) analysis. They showed that the FT4 and FT3 results significantly increased with the HBT test. Thereupon, they applied gel filtration chromatography and revealed that the FT4 and FT3 peaks overlapped in the IgM elution time. They suggested that the non-specific antibodies may have been somehow activated in the test reagent due to the possibility of absorption of IgM antibodies. We can consider this hypothesis as one of the potential causes of the increase in our LH levels.

As reported by Ellis et al. [14], in a 53-year-old male case, the Beckman Coulter Access analyzer showed that LH, FSH, growth hormone (GH) and to a lesser extent prolactin (PRL) values measured after HBT pretreatment were increased and evaluated this result as spurious. The reagents used to prove heterophile Ab interference may cause interference in some immunoassay systems. This situation seems to be due to the possible structure of the capture and detection antibodies in the analysis systems. Specifically, the presence of solid-phase goat anti-mouse monoclonal antibody complexes in these tests makes the use of blockers containing murine (mouse-derived) components (such as HBT) unsuitable. This highlights the need for alternative validation strategies to HBT in cases of suspected heterophilic interference in immunoassay tests containing an anti-mouse component.

In addition, HBTs containing blocking agents do not guarantee complete blockade, and this does not mean that there is no interference [3]. HBT containing monoclonal mouse anti-human IgM adsorbs IgM [13]. The antibodies in the serum sample may not have been recognized by the blocking antibody in the HBT or the blocking feature of the tubes may have been ineffective if the antibodies were at a very high concentration [3].

Due to the design of the kits used in the study, the HBT reagent may not have produced the expected improvement in LH measurements. Heterophilic Ab interference can lead to false LH results [14,15] and other test results measured incorrectly by immunoassay methods [13,15,16,17]. Although false high values are more common due to heterophilic Ab, false low (cortisol) results are also seen [2]. There are reports showing that the analyte measured after HBT pretreatment increases [3] as well as decreases [15,18,19]. In some analyses such as thyroid function tests [20], estradiol (E2) [21], cardiac troponin I (cTnI)-cardiac troponin T (cTnT) [22], FSH [16], FT4 and cortisol [2], HBT treatment was ineffective in eliminating the interference.

In a case with premature thelarche and elevated basal LH levels, the basal and stimulated LH values decreased after HBT pretreatment with two different immunoassay methods, thus showing the presence of heterophile Ab in the serum sample. The falsely high LH concentration due to these antibodies led to a false diagnosis of central precocious puberty and unnecessary treatment planning [23]. In a prospective study analyzing 5310 patient samples, it was shown that there was interference for LH, FSH and TSH tests in 28 patients (0.53%). Interference was detected after treatment with heterophile blocker in 23 of 28 samples (82%) [24]. There are also studies that detect falsely high LH and multiple hormones analyses due to high RF interference using HBTs [2]. In pediatric patients, transient RF positivity—often unrelated to autoimmune disease—may act as a source of immunoassay interference. Therefore, in cases where hormone results such as LH are discordant with clinical findings, the potential contribution of RF–related or heterophile Ab interference should be considered. Our patients may have experienced a temporary increase in RF levels, possibly due to a past infection.

There was no statistically significant difference between the serum LH values measured with Roche Cobas e601 without HBT pretreatment and after HBT application (*p* > 0.05). When we compared the serum LH values analyzed after HBT pretreatment in Roche Cobas e601 and Beckman Coulter autoanalyzers, we found that although the LH values measured in Roche Cobas e601 were lower, there was no significant difference (*p* = 0.76). There were extreme values in the measurement results in the Beckman Coulter device after HBT pretreatment, and therefore we evaluated that the difference was not statistically significant. When a change occurs after pre-treatment with HBT, it is likely that an interference factor is present, but the absence of a change does not rule out the possibility of interference.

The Roche Cobas e601 analyzer performs analysis with the electrochemiluminescence immunoassay: ECLIA method, while the Beckman Coulter analyzer performs analysis with the chemiluminescence immunoassay: CLIA method. Both are very similar methods in terms of method. However, when the LH reagent contents are examined, it is seen that they are designed differently by each manufacturer.

The manufacturer mentioned in the limitations section that there is a possibility of interaction with heterophile antibodies. Moreover, it was stated that interferents such as HAMA, RF, anti-goat antibody and alkaline phosphatase (ALP) may cause erroneous results in the analysis [8]. HAMA, RF and heterophile antibodies may affect LH assays [3].

Beckman Coulter reported in a customer letter (CASE-2023-02287445) that two of the samples we sent had interference with ALP and that the results had decreased after AP Mutein and Scavenger ALP blocker. He shared his view that these interfering agents were the likely cause of our falsely high Access hLH results. He suggested that we record this information in the patient’s clinical file and carefully interpret future results of this patient in light of this potential risk. Since our patients were in the pediatric age group, an elevation in ALP is a physiologically expected process; moreover, it exposes our results to the risk of ALP interference.

In a study on ALP interference, false low results were detected in the Beckman unconjugated estriol (uE3) test due to anti-ALP antibodies, and on the other hand, it led to false positive prenatal screening test results in the risk calculation of trisomy 21 and trisomy 18 [25]. Like false uE3 results due to antibodies against reactive alkaline phosphatase, false results have been reported in adult patients with different tests [26,27,28]. In this respect, we suggest that it would be useful for the manufacturer to evaluate different options instead of signal amplification with ALP in the kit reagent design. In an interesting study, LH and FSH values measured abnormally high (Beckman Coulter DxI autoanalyzer) in a woman during menopause transition were within the normal reference range after HBT treatment. After the presence of heterophile antibody was proven, it was desired to analyze its type, so it was subjected to pre-incubation with pure goat IgG. In the repeated measurement, gonadotropin levels were found to be significantly lower [15]. In case of suspected heterophile antibody interference, it is important to first evaluate antibodies formed against kit reagent components. Although there was no known goat exposure in this case, it is frequently encountered in the community, especially in those engaged in animal husbandry. We are aware that individuals engaged in animal husbandry frequently attend our hospital; however, it is uncertain whether this exposure was systematically assessed in our patient population.

Although less common, macro-LH, like macroprolactin, has also been described [29,30,31]. In a case report presented by Umemori et al. [29] reported that LH formed a complex with immunoglobulin G (IgG) autoantibodies, indicating the presence of macro-LH, and therefore, the clearance of LH from the blood was delayed and remained high. In this study, the presence of IgG Ab was detected with anti-IgG Abs and Protein G addition test, and this was confirmed by high-performance liquid chromatography (HPLC) analysis [29].

Inconsistent sample results can be obtained with different analytical methods. Because analytical errors often depend on the characteristics of the method. In particular, report of interference from ruthenium (Ru) signals have been reported in the streptavidin-biotin separation method and the Roche Cobas autoanalyzer reagent [32]. In two case reports presented in 2024, where kits containing modified Ru sulfonate complexes were used, it was found that thyroid function tests (FT4, FT3 and TRAb) were falsely high. However, in measurements made with existing (old version) Ru-containing kits, FT4 and FT3 values were within the reference range. It was concluded that antibodies targeting the Ru sulfonate complex in the new kit content developed by the manufacturer were developed [13].

In cases where heterophile Ab interference is suspected, there are reports that comparing the analysis results with a different immunoassay method, linearity testing with sample dilution, protein A pretreatment and the use of heterophile blocking tubes (HBTs) are beneficial. However, no method alone is sufficient [1].

Serial dilution of the sample is the cheapest and simplest troubleshooting approach when interference is suspected. However, some samples resist dilution due to matrix effects and components. It has been shown to be unsuitable for free thyroid hormone assays. Moreover, it may be misleading in the evaluation of samples containing heterophilic Ab [2]. We did not perform serial dilution of the sample for LH analysis because it may be misleading.

We avoided using the polyethylene glycol (PEG) precipitation method because it was predicted to be ineffective in Beckman Coulter analyzers [1].

Liquid chromatography–tandem mass spectrometry (LC–MS/MS) is considered the reference method for steroid analysis in pediatric patients [3]. In a case report published in 2025, estradiol levels in a 10-year-old girl were found to be elevated and inconsistent across three different immunoassay platforms, and the presence of analytical interference was subsequently confirmed using LC–MS/MS [21]. As the use of LC–MS/MS to resolve suspected assay interference becomes more widespread in clinical laboratories, reliance on immunoassays for steroid measurements—and consequently the impact of immunoassay-related interference—may be reduced [3].

In addition, several factors may contribute to inconsistencies between laboratory results, including patients’ demographic characteristics, the presence of unrecognized autoimmune disease, current or previous viral and/or bacterial infections, RF positivity, the increasing use of antibodies and recombinant proteins as diagnostic and therapeutic agents, vaccination practices, and exposure to domestic animals [1]. Moreover, exposure to acute or chronic parasitic or fungal infections may also lead to assay interference through elevated levels of certain antibodies. Although these factors are most frequently reported in adult populations, they may also be relevant in the pediatric age group. False-positive or false-negative LH results can have substantial clinical consequences in the evaluation of puberty-related disorders, particularly central precocious puberty. Falsely elevated LH levels may lead to premature diagnosis, unnecessary imaging studies, and unwarranted initiation of gonadotropin-releasing hormone analog therapy, exposing patients to avoidable psychological and medical burden. Conversely, falsely low LH results may delay or obscure the diagnosis, resulting in missed opportunities for timely intervention during a critical developmental window. In the presence of assay interference, such as heterophile antibodies, discordance between biochemical findings and clinical presentation should prompt careful reassessment to prevent misclassification and inappropriate management.

### Limitations of the Study

The main limitation of our study is that alternative methods for detecting heterophile antibody interference were not included. Due to the limited serum volume typically available from pediatric patients compared with adults, only a single interference detection approach could be applied. Because of technical constraints, patient samples could not be analyzed using high-performance liquid chromatography (HPLC), LC–MS/MS, or gel filtration chromatography. For this reason, the specific type and titer of the heterophile antibodies potentially responsible for the observed interference could not be identified.

We concluded that while HBTs were ineffective in detecting heterophile antibody interference in LH testing, the study underscores the complexity of interference in pediatric hormone assays and highlights the need for further investigation into alternative methods to ensure reliable test results in this age group. Consistent with this, we demonstrated that HBT pretreatment did not alter hormone assay results obtained using Roche Cobas diagnostic systems. Furthermore, pediatric age-specific reference intervals have not been established for hormone assays on the Beckman Coulter UniCel DxI platform, which represents an additional limitation and highlights the need for further studies in this area.

## 5. Conclusions

In conclusion, this study contributes to a more refined understanding of heterophile antibody interference in LH immunoassays, particularly within the pediatric and adolescent population. By demonstrating how assay interference can generate clinically misleading LH results that are discordant with physical findings, our findings underscore the importance of integrating laboratory vigilance with clinical judgment in the evaluation of puberty-related disorders. These results reinforce the need for systematic consideration of immunoassay interference when LH values are incongruent with the clinical phenotype. Future research should prioritize the development of more reliable and standardized interference-blocking strategies, as well as the establishment of pediatric-specific assay validation and decision thresholds that account for age- and puberty-dependent hormonal variability, in order to improve diagnostic accuracy and patient management.

## Figures and Tables

**Table 1:** Age distribution by gender.

Age (Years)			
	Girl Child (n: 38)	Boy Child (n: 10)	Total (n: 48)
Mean ± SD	10 ± 2.557	11.80 ± 1.033	10.38 ± 2.429
Median (Min–Max)	10 (8–13)	12 (10–13)	10.50 (8–13)

SD: Standard deviation; Min: minimum; Max: maximum.

**Table 2:** LH values before and after HBT pretreatment according to age and sex measured on two different immunoassay analyzers.

	Age	BC	BC/HBT	RC	RC/HBT
Pediatric female patient	8	0.93	8.55	1.16	1.23
	8	0.67	2.77	0.67	0.77
	8	3.57	7.2	4.05	4.05
	8	0.36	1.98	0.39	0.52
	8	1.82	1.57	2.34	2.37
	9	3.39	15.74	0.1	0.1
	9	1.05	1.06	1.47	1.56
	9	0.74	16.77	0.96	1.02
	9	1.11	1.91	1.35	1.55
	9	0.7	1.4	0.42	0.38
	9	1.11	0.24	0.35	0.37
	9	0.32	0.3	0.45	0.46
	9	0.77	1.25	1.33	1.43
	9	1.3	1.8	1.25	1.21
	9	0.37	0.4	0.47	0.52
	10	1.5	2.27	2.08	2.09
	10	0.93	0.78	1.17	1.24
	10	1.48	3.04	1.6	1.81
	10	0.84	1.0	1.19	1.16
	10	0.56	0.62	0.87	0.8
	10	1.23	0.44	0.34	0.29
	10	0.32	0.35	0.46	0.45
	10	0.87	1.02	1.33	1.24
	10	1.13	48.93	1.42	1.49
	10	0.6	0.65	1.14	1.07
	11	4.75	4.57	6.14	6.55
	11	1.62	1.54	2.64	2.32
	11	0.34	0.36	0.54	0.57
	11	0.33	0.37	0.7	0.69
	11	1.98	1.77	2.67	2.71
	11	1.47	1.55	2.26	2.18
	11	1.51	1.31	1.91	1.94
	12	2.94	2.64	3.78	3.68
	12	1.77	5.38	2.11	2.31
	12	3.62	3.36	4.6	4.78
	12	1.71	1.5	2.6	2.46
	13	3.19	3.72	4.2	4.19
	13	1.17	1.4	1.66	1.76
Pediatric male patient	10	0.66	5.52	0.99	0.97
	11	0.87	2.73	0.92	0.99
	11	0.96	0.77	2.31	1.95
	11	1.11	1.12	2.01	1.88
	12	0.61	0.61	0.86	0.95
	12	1.56	1.29	2.01	1.96
	12	0.37	0.4	0.67	0.66
	13	1.2	0.93	1.46	1.5
	13	0.56	0.57	0.91	0.93
	13	0.87	1.24	1.46	1.38

BC: Beckman Coulter; HBT: Heterophilic blocking tube; BC/HBT: Analysis with BC after HBT pretreatment; RC: Roche Cobas; RC/HBT: Analysis with RC after HBT pretreatment. Measurement range: 0.3–250 mIU/mL (BC). Girls; 6–10 years: <0.1–3.1; 11–13 years: <0.1–11.9 Boys; 6–10 years: 0.1–1.4; 11–13 years: 0.1–7.8 (mIU/mL, RC).

**Table 3:** Analysis of LH values before and after HBT pretreatment on Beckman Coulter UniCel DxI 800 autoanalyzer and Roche Cobas e601 autoanalyzer.

Beckman Coulter UniCel DxI 800			
LH (mIU/mL)	Before HBT	After HBT	Test statistics
Mean ± SD	1.31 ± 0.99	3.47 ± 7.51	*p* = 0.01
Median (Min–Max)	1.08 (0.32–4.75)	1.40 (0.24–48.93)	
**Roche Cobas e601**			
**LH (mIU/mL)**	**Before HBT**	**After HBT**	**Test Statistics**
Mean ± SD	1.62 ± 1.24	1.64 ± 1.27	*p* = 0.27
Median (Min–Max)	1.33 (0.10–6.14)	1.31 (0.10–6.55)	

SD: Standard deviation; Min: minimum; Max: maximum.

**Table 4:** High differences between LH values before and after HBT pretreatment measured with Beckman Coulter UniCel DxI 800.

Before HBT	After HBT	Increase (Times)
3.39	15.74	4.64
0.87	2.73	3.14
0.93	8.55 *	9.19
0.74	16.77 *	22.66
0.67	2.77	4.13
1.77	5.38	3.04
3.57	7.2	2.02
0.66	5.52 *	8.36
1.13	48.93 *	43.30

* Very high LH values (mIU/mL).

**Table 5:** Comparison of LH values before HBT and after HBT pretreatment in two autoanalyzers.

LH (mIU/mL)	Beckman Coulter UniCel DxI 800	Roche Cobas e601	Test Statistics
Before HBT pretreatment			
Mean ± SD	1.31 ± 0.99	1.62 ± 1.24	*p* < 0.001
Median (Min–Max)	1.08 (0.32–4.75)	1.33 (0.10–6.14)	
After HBT pretreatment			
Mean ± SD	3.47 ± 7.51	1.64 ± 1.27	*p* = 0.76
Median (Min–Max)	1.40 (0.24–48.93)	1.31 (0.10–6.55)	

SD: Standard deviation; Min: minimum; Max: maximum.

## Data Availability

The data underlying this article are available in the article. If needed, please contact the corresponding author. The email address is drzeki@yahoo.com.
